# Early Maternal Influences on Stress Circuitry: Implications for Resilience and Susceptibility to Physical and Mental Disorders

**DOI:** 10.3389/fendo.2014.00244

**Published:** 2015-01-14

**Authors:** Nikolaos P. Daskalakis, Rachel Yehuda

**Affiliations:** ^1^Traumatic Stress Studies Division, Department of Psychiatry, Icahn School of Medicine at Mount Sinai, New York, NY, USA; ^2^Mental Health Patient Care Center, James J. Peters Veterans Affairs Medical Center, Bronx, NY, USA; ^3^Laboratory of Molecular Neuropsychiatry, Department of Psychiatry, Icahn School of Medicine at Mount Sinai, New York, NY, USA; ^4^Department of Neuroscience, Icahn School of Medicine at Mount Sinai, New York, NY, USA

**Keywords:** early life stress, HPA axis, vulnerability, resilience, maternal

Experiences in early life result in enduring and sometimes permanent differences in how the central nervous and endocrine systems function. These changes are referred to as developmental programing, and have numerous consequences for susceptibility to physical and mental disorders, which involve central nervous and endocrine systems. These effects may depend on genetic factors, but more importantly, involve epigenetic mechanisms, which can change the function of the gene independently of genotype. Environmentally induced epigenetic changes alter gene expression and function, and their outcome leads to adaptive or maladaptive expressions, depending on the specific environmental context of the individual (1).

In this Frontiers Research Topic “Programing the hypothalamic pituitary adrenal (HPA) axis by early life experience: mechanisms of stress susceptibility and adaptation,” we have assembled a comprehensive selection of original research, mini reviews, and review articles that describe prenatal and postnatal maternal influences on stress response systems in offspring. This is an emerging and critical area in neuroendocrine science as it is becoming increasingly clear that much of the variance associated with biological alterations in physical and mental disorder may be accounted for by maternal history and experiences in the womb or in early postnatal life. While such influences have been broadly acknowledged, until recently, it has been difficult to measure the effects of these maternal influences on offspring. Epigenetic marks can be measured and provide a quantification of relevant early environmental influences. Carefully designed studies can then parse out the origin of early influences.

In the opening paper of the volume, Daskalakis and Yehuda systematically review the methodologies employed to study epigenetic effects of early adversity (2). They focus on studies of the most studied genomic region in human stress-related diseases, the exon 1_F_ promoter of the glucocorticoid receptor (GR) gene. The discussion is meant to present a series of issues that influence quantitative methylation analyses (method, tissue type, region of interest). It is important in an emerging field to discuss such issues since they can result in variable results from laboratory to laboratory. The authors make the important point that there may be site-specific accommodations of different adversity factors in different CpG sites within a promoter-based CpG island, leading to a greater complexity of data analysis and interpretation than is currently the standard. Finally, this review paper also includes unpublished data that extend the transgenerational effects previously observed in this region (3) by demonstrating site-specific effects based on maternal and/or paternal PTSD.

After this focused review, two review papers provide an overview first of the effects of early adversity on major depression, as an example of a critical psychiatric outcome, and second on the effects of early adversity on metabolic outcomes. Hornung and Heim (4) summarize recent human studies implicating gene by early life stress interactions in vulnerability and resilience for depression. The authors chose the term “intermediate phenotype” throughout the paper in order to emphasize the mediational character of some phenotypic features, which result from gene by environment interactions and may lead to depressive symptoms. Maniam et al. (5) using animal and human data support that early adversity induced maladaptation of the HPA axis will have negative impact on energy metabolism in the presence of chronic stress or unfavorable metabolic conditions in later-life leading to vulnerability to metabolic diseases. To this end, authors have attempted to review the relevant literature, with attention paid to findings dealing with metabolic/body weight measures. They further propose a mechanism whereby the cross-talk between HPA axis and altered glucocorticoid (GC) metabolism in the liver (by 11-beta hydroxysteroid dehydrogenase Type 1) may likely mediate the metabolic outcomes of early life stress.

Following these overviews, papers were divided according to the developmental time window in which adversity was present. Two original research articles describe the effects of prenatal maternal exposures to alcohol and GCs on stress-related neurocircuits. Raineki et al. (6) reveal complex sexual dimorphisms by providing a comprehensive (constrained principal component) analysis of the neural activity (by *c-fos* expression) in amygdala, hippocampus, medial prefrontal cortex, and paraventricular nucleus of hypothalamus in response to acute stress in male and female offspring of dams exposed to alcohol or two control conditions in gestation (gestation day 1–21), which where additionally exposed to a 10-day chronic mild stress paradigm in the peripubertal period. Borges et al. (7) find that prenatal maternal exposure to dexamethasone (gestation days 18 and 19) increased the activity of two regions that belong to the mesopontine cholinergic system (laterodorsal and pedunculopontine tegmental nuclei) associated with enhanced behavioral stress vulnerability (anxiety behavior including fear-associated ultrasonic calls). The review by de Kloet et al. (8) complements the Borges paper by focusing on the impact of perinatal GC exposure on the developing HPA axis and the balance between GR and mineralocorticoid receptor. The authors review animal and also clinical research on perinatal disturbances by GC administration and possible intervention strategies.

Postnatal stress in rodents also exerts lasting effects on the stress system as well as on other brain systems. Daskalakis et al. (9) provide original data on strain-dependent differences in the immediate effects of maternal deprivation on the neonatal HPA axis response. While maternal deprivation causes a disruption of the neonatal stress hyporesponsive period in both mice strains studied, one strain was more affected calling for future studies on later-life consequences of early postnatal stress based on genetic background. Loi et al. (10) describe how hippocampal neurogenesis is affected by early life stress in rodents in an age- and sex-dependent manner, and provide novel data showing that normalization may be possible through GC-based interventions during critical stages of brain development. Brief treatment with mifepristone, a GR antagonist, on postnatal days 26–28 protected female rats against the effects of maternal deprivation (postnatal day 3) on neurogenesis markers in dentate gyrus. This finding is interesting because mifepristone is currently being tested in a clinical trial for post-traumatic stress disorder (11), a condition, which has also been linked with maternal PTSD (12), and childhood trauma (13). Loi et al. also speculate about the implications of findings in animal models of perinatal stress for the study of human brain development and vulnerability to psychopathology. Cinini et al. explore the effects of adversity in an early post-weaning period on hippocampal neurogenesis (14). The authors showed that social isolation in young (8–10 months old) non-human primates (marmosets) produced anxiety-like behaviors, elevated cortisol levels, and reduced neurogenesis.

Long-term effects of perinatal stress experiences are partially mediated by mother–pup interactions (15). How these interactions influence offspring development is summarized in the mini-review by Lucion and Bortolini (16) where the special role of genes, epigenetics, imprinting, neurohormones, and learning is outlined. The review by Rincon-Cortés and Sullivan continues on the same theme (17). It is explained that the development of rat odor aversion develops after postnatal day 10, when maternal absence can increase GCs and subsequently activate a fear-related amygdala-dependent neurocircuit. The effects of maternal contact are also explored in the mini-review by Stamatakis et al., who summarize findings from their new model of neonatal learning (18). In order to investigate the effects of the early maternal environment, rat pups either receive the expected reward of maternal contact or are denied this reward on postnatal days 10–13. The two training conditions have differential consequences on brain activity, HPA axis function, cognition, and stress-related behavior.

The final paper, a human study, by Bader et al. (19) explores transgenerational effects of parental trauma in offspring. The findings replicate and extend previous findings regarding urinary cortisol levels in Holocaust survivor offspring (20–22). Authors observed that the low cortisol levels in association with maternal PTSD are additionally associated with maternal age at Holocaust exposure (a parallel effect on cortisol in offspring that is not related to maternal PTSD).

To conclude, the articles of this Research Topic have highlighted the importance of investigating the biological effects of early stressful factors of maternal origin on stress circuitry in order to comprehend resilience and susceptibility to physical and mental disorders. The introduction of modern methodologies (e.g., epigenetic analyses) can give traditional neurobiological findings (e.g., neurogenesis markers) a molecular understanding.

## Conflict of Interest Statement

The authors declare that the research was conducted in the absence of any commercial or financial relationships that could be construed as a potential conflict of interest.
